# The Birkenhead drill suggests ‘women and children first': government and society’s reversal of the drill during the COVID-19 pandemic, left children last and cannot be allowed to continue

**DOI:** 10.1136/postgradmedj-2020-137991

**Published:** 2020-10-17

**Authors:** Joe Brierley, Vic Larcher

**Affiliations:** Paediatric Bioethics Centre, University College London Great Ormond Street Institute of Child Health, NIHR Great Ormond Street Hospital Biomedical Research Centre, London, WC1N 1EH, UK; Paediatric Bioethics Centre, University College London Great Ormond Street Institute of Child Health, NIHR Great Ormond Street Hospital Biomedical Research Centre, London, WC1N 1EH, UK

**Keywords:** Ethics (see Medical Ethics), Paediatrics, Health services administration & management, Health policy

## Abstract

The Birkenhead drill states that in the time of crisis, the correct action is to prioritise the weakest and most vulnerable, in that example, women and children. Ethically this has been well analysed in terms of the intrinsic value of the human versus any utilitarian calculus of worth to society’s function. We do not attempt to re-analyse this but do note that standard pandemic planning often disadvantages the weak and vulnerable in terms of allocation of resources to those with a greater chance of functional survival. We more argue from a debt that society owes its children in terms of the sacrifices they have made in terms of school, social life, healthcare and overall welfare during the pandemic from which they were at markedly less risk than adults. Society owes a debt to its young, and this on top of pre-existing commitments to the them that most nations fail to realise, calls for prioritisation of children and young people’s issues as society rebuilds. The effects of poverty and systemic racism on many children must be tackled; so too the existential threats of climate change and pollution. COVID-19 provides a once in a generation opportunity to create a kinder, fairer society. Early signs are not good: Pub re-opening prioritised over school re-opening; no significant investment in children’s services or women’s health, a significant determinant of children’s welfare. We highlight the way COVID-19 has, and continues, to harm children and argue that the contemporary erosion of the Birkenhead principle is simply amoral.

*So they stood an’ was still to the Birken’ead drill, soldier an’ sailor too!* ^1^

A long-established response to existential threats posed by adverse events or natural disasters is the obligation to protect the weak and vulnerable. This response, characterised in maritime tradition as ‘Women and children first’, finds expression as the Birkenhead drill of Kipling’s poem ‘Soldier an’ Sailor Too’.^1^ The poem not only honours the courageous behaviour of those who sacrifice themselves for others but also identifies groups who, because of their vulnerability and instrumental value, require special the moral consideration that requires such selfless behaviour. Just as contemporary views of Kipling and the sentiments he expressed have changed, so too has our perspective on those, if any, who merit special moral consideration. For example, ethical advice produced in advance of a predicted Influenza pandemic was founded on the central principle of equal concern and respect for all, making no specific provision for especially vulnerable groups other than could be adduced by application of the principle of reciprocity.^2^

Nevertheless, the concept continues to find resonance in the current COVID-19 pandemic, where healthcare resources have been stretched, and the lives of healthcare professionals and others providing essential services have been lost, like the troops on the Birkenhead.^3^

The initial response to the pandemic focused on saving as many lives as possible by protecting the scarce resources necessary to provide life-sustaining therapy in intensive care.^4^ At that stage, much of the ethical guidance concerning children centred on the acute management of COVID-19,^5^ but acute severe infection in children has been thankfully rare with <2% requiring ICU admission and thankfully few deaths reported worldwide.^6^ In contrast, the severe disease and concomitant higher mortality seen in specific subgroups such as the elderly and the BAME population have affected children due to mortality and morbidity in their family and community. The exception in childhood has been the emergence of the post-inflammatory condition, PIMS-TS or MIS-C, which has affected some children, with an increased risk on the BAME community to which we will return.^7 8^

As indicated, and perhaps because of the uncertainly over which groups may be most affected, generic ethical frameworks for the acute management of pandemics do not expressly grant greater moral claims for preferential treatment to any particularly vulnerable group, for example, children. This stance does seem counterintuitive to many^9^ and is undoubtedly discordant with the Birkenhead drill.

Nevertheless, the current crisis has continued to highlight several secondary issues that impact on children both now, but also in the future.^10 11^ It is clear that women, in their roles as parents, unpaid carers or breadwinners, often at an economic disadvantage, are suffering from the socio-economic effects of the pandemic. This impacts on their ability to fulfil their many essential roles, and thus affects children.^12^

As well as the predictable effects on contemporary child-health services,^13^ there have been less predictable consequences; some generic others specific to children (table 1). Few have been identified explicitly in ethical guidance proffered to date.

Despite the initial mild nature of paediatric COVID-19 infection, the far-reaching impact of the pandemic on the young, demands that we do not limit concerns to the minimisation of adult mortality and economic recovery while ignoring the broader effects on children. Despite current reconsideration of Kipling due to his association with Empire, we argue that the principle his poem espouses remains a morally defensible action based on a persisting notion of the ‘protection of the vulnerable’ in a disaster setting. We caution against any of erosion of the Birkenhead principle but will illustrate how that is precisely what has and continues to happen during the COVID pandemic.

*During this pandemic, children, families and child-health professionals are being or will be, affected in several ways (* *table 1*)

**Table 1:** Impact of the COVID-19 pandemic on children, families and child-health professionals

Children with acute COVID-19 infection ± alternative infective aetiologies admitted to wards, high-dependency or intensive care units. Children with chronic conditions deteriorate either by the direct infective effect of COVID-19 or compromise to usual treatments, for example, need for reduced immunosuppression to avoid the risk of C-19. Children with complex care needs in the community have reduced service (depleted support staff, diverted or triaged resources or lack of usual access to acute care). Children requiring elective surgery deteriorate due to disease progression, for example, congenital heart disease or gastro-oesophageal reflux associated aspiration. Children with a new incident/continuing illness have limited access to all levels of inpatient facilities—depleted specialist and general paediatric care, community support (GP, community nursing teams) (see 7). Social isolation and school closure leading to mental health issues; lack of regular teenage interaction; concerns for children on welfare or in need of child protection, that is, risk of non-accidental trauma, neglect or other abuse. Healthcare professionals infected, thereby posing a threat to patients, or be contacted isolated and/or unable to work due to bereavement/caring for dependent relatives.

A wide range of standard treatment has been compromised during the COVID elective healthcare lockdown, for example, spinal fusion pathways, live-related transplants, suboptimal/amended chemo-radiotherapy regimens for malignancies. There have been shortages of essential medicines, imaging and radiotherapy treatment, including ventilation circuits required to keep children on long-term ventilation at home alive.^14^ Innovative, speculative or costly therapies can seem inappropriate when others are dying due to lack of ventilators or personal protective equipment (PPE); examples include long-term ventilation for time-limited trials of brain injury recovery, experimental neurometabolic encephalopathy protocols, and palliative ventilation for advanced respiratory failure.

There have been concerns about the impact of widespread cancellation of outpatient appointments on ongoing care, and deaths have occurred because parents were reluctant to bring sick children to A&E departments secondary to fear of exposure of themselves and their child to COVID.^15^ Disruption of community-based immunisation programmes, developmental follow-up, and child-health surveillance have been identified as significant concerns. It has been estimated that 80 million under 1-year-old children worldwide are at risk of vaccine-preventable diseases.^16^ Together with the considerable disruption of other proven interventions, the effect on global child mortality is potentially catastrophic.^17^

Compassionate and holistic complex or end-of-life care for terminally ill and frail children has become increasingly challenging, with remote consultation technologies supplanting face-to-face human contact with all its nuance.

The reduction in maternal and child-health services produced by COVID-19, including the ongoing effect of widespread delayed treatments, will potentially have a significant impact in terms of excess child deaths and morbidity.

Healthcare systems are not the only determinant of child-health. Widespread and prolonged social isolation places significant strain on families and their coping strategies; increased levels of anxiety have already been reported in some children and a considerable proportion of parents.^18^ Isolation heightens the risk of domestic abuse and increases the impact of parental drug and alcohol abuse and other mental health issues. Child-health teams must be alert to the heightened possibility of all categories of child abuse, maltreatment and neglect whether novel or pre-existing.^19^ Additionally, school/nursery closures, mean the loss of crucial social and developmental resources, and safe and secure support systems for the vulnerable. Despite the provision for continued schooling for children of essential workers and those who were vulnerable, there was only limited uptake of this facility.

Moreover, there is little evidence to suggest that school closure has a significant impact on reducing deaths in previous coronavirus pandemics.^20^ Despite attempts to alleviate detrimental educational effects by distance learning programmes, not all will have the facilities or domestic structure to benefit. It is likely that childhood poverty, with its concomitant effects on health and well-being, will increase as not all families will benefit from welfare programmes or income support schemes. Families whose incomes dropped as a result of work restrictions have struggled to feed their children,^21^ with a worsening of already appalling childhood malnutrition rates secondary to austerity.^22^ Paradoxically, prolonged social isolation and financial difficulties may have increased the risks of childhood obesity, and its long-term sequelae, due to imposed physical inactivity and poor diet.^23^ As with healthcare professionals, illness and issues with dependent relatives restricted the number of frontline welfare staff at a time of greatest need, the effects are only now being visualised.

The impact of the pandemic on the mental health of not just children, but of professionals, is a significant concern. Inability to deliver the usual standards of care and the need to make distressing existential decisions in a short time-frame without adequate support is a risk or moral distress and injury, with secondary burnout in a wide range of professionals.^24^ These issues must be addressed if children are to return to a caring healthcare system after the pandemic has passed.

The effects of exposure to mortality in younger children, with daily death rates reported in the media, and isolation from older relatives to decrease mortality risk on grandparents and others may well engender a less secure view of their existence. For teenagers/adolescents, the uncertainty over exam performance/grading and progression is unprecedented. The lack of usual social interaction with peers to provide support, the development of physical relationships and to just hang out with (current vernacular notwithstanding) is troubling at such a critical stage.

This pandemic is having and will have, both overt and covert deleterious effects on the health of children. A Tsunami of documents/information—ethical and process driven—have appeared from scientific societies, governments and others, and more rapidly than ever via social media; most focused on the care of adults. However, the effects on paediatric practice, both known and as yet unknown, cannot be ignored.

All members of society, including children, have been asked to make unprecedented sacrifices during this pandemic. The natural rights of many citizens, including some enumerated in the UN Convention of the Rights of the Child, were compromised, insofar as it was thought proportionate to do so. In the acute situation, welfare rights had to take precedence over liberty rights, for example, concerning lockdown. The usual contractual basis of personal healthcare, based on the consideration of individual best interests and clinical need was, to some extent, superseded by a more utilitarian outcome-based model, applied when resources are scarce. It is not clear whether this model should be used outside the acute situation, that is, during recovery and whether it is proportionate to do so. Children may, by reason of incapacity, be unable to exercise liberty or choice rights. Still, they do have both welfare and protection rights which, as is evident from a consideration of the examples above, may be compromised. They have a right to an open future,^25^ though whether that is one that maximises their welfare, and their choices or provides one which a reasonable person might want is an open question.^26^ But children, unlike adults, have no voice over the proportionality of the measures introduced or on the impact of those interventions on their current and future well-being; they may not be included in discussions even when deemed competent to do so. As ever, they need those who will advocate on their behalf.

Children from poorer socio-economic backgrounds are already adversely affected with respect to their future health, well-being education and social mobility. These inequalities are worse in children from BAME backgrounds, and COVID-19, and its socio-economic consequences, has further exacerbated these adverse effects—so that children from this background suffer double jeopardy regardless of whether they develop COVID themselves. The Black Lives Matter movement has started, during the period of COVID-19, to force an overdue address of systemic racism and inequality throughout many societies and services. The effects of this pervasive injustice on children and their families affect every aspect of children’s lives, whether education, life opportunities or health and the resultant harm is both long-standing and recurrent.^27^

Even before COVID-19, children’s rights were inadequately recognised and prioritised in most countries; a situation only worsened due to the severe economic fallout of the pandemic. Arguably, the most critical issue facing our children is climate change and associated pollution, and there is an unprecedented opportunity to use COVID-19 to make the sustainable changes required.^28^ The necessity to change the materialistic, consumerist society into one that strives for a secure future for generations to come has never been more apparent. Nor arguably has this been more achievable given the opportunities afforded by lockdown-induced remote working, such as reduced car and aviation use.

Sadly, this hoped for better, kinder, post-COVID world appears increasingly unrealistic as the rush to re-open the economy takes precedence over everything else. The inadequate consideration of children’s issues as society emerges from COVID-19 is well illustrated by the rush to re-open pubs, but not schools.^29^ There is, indeed, a global accountability crisis for women’s, children’s and adolescent’s health,^30^ heralding a tragic contemporary reversal of the Birkenhead drill.

## CONCLUSION

We accept that any plea for treating children differently from any other vulnerable individuals should be ethically justified, with due consideration of the principles of minimising harm, and distributive justice. Any claims made on behalf of children should be reasonable, accountable, transparent and proportionate, but the principles of justice and reciprocity require that any claims that society makes on children fulfil the same criteria. Most children will recover from COVID-19, but their future will be affected by the socio-economic consequences of the pandemic in a way that differs from adults. Their right to an open future necessarily entails that their future interests cannot be ignored. Governments, policymakers and healthcare professionals must not allow reversal of the Birkenhead drill to leave children last.
